# Mixed Warm and Cold Autoimmune Hemolysis in an Elderly Patient With Sickle Cell Disease and End-Stage Renal Disease on Hemodialysis: A Diagnostic and Therapeutic Challenge

**DOI:** 10.7759/cureus.92352

**Published:** 2025-09-15

**Authors:** Nicholas Lorenz, Justin Brown-Gnarra, Marc Graham, Kelvin Bray

**Affiliations:** 1 Orthopaedic Surgery, Lake Erie College of Osteopathic Medicine, Bradenton, USA; 2 Medicine, Lake Erie College of Osteopathic Medicine, Jacksonville, USA; 3 Internal Medicine, Baptist Medical Center Beaches, Jacksonville, USA

**Keywords:** anemia, auto immune, mixed aiha, sickle cell disease complications, sickle cell disease (scd)

## Abstract

Mixed autoimmune hemolytic anemia (AIHA), defined by the simultaneous presence of warm and cold autoantibodies, is a rare and severe complication in patients with sickle cell disease (SCD), particularly in older adults. This case describes a male in his late 60s with homozygous SCD and end-stage renal disease (ESRD) who developed mixed AIHA, presenting with profound anemia and laboratory evidence of hemolysis. The advanced age and comorbidities complicated both diagnosis and management, with transfusion support hindered by alloimmunization. Despite these challenges, corticosteroid therapy and multidisciplinary care contributed to clinical stabilization. This case underscores the need for heightened clinical suspicion and tailored management strategies for mixed AIHA in elderly SCD patients.

## Introduction

Sickle cell disease (SCD) has historically been considered a condition of childhood and young adulthood, due to its significant morbidity and previously limited life expectancy. However, with advances in supportive care, hydroxyurea therapy, infection prevention, and overall disease management, the median survival for SCD patients in the United States has increased to beyond age 40 [1]. Survival into the sixth and seventh decades of life is confirmed but remains relatively rare, as the majority of patients do not reach age 60, and life expectancy remains 20 to 30 years shorter than that of the general population, even in high-resource settings [2]. Despite these gains, older adults with SCD remain a small but vulnerable subset, often burdened with cumulative organ damage, including renal failure, cardiopulmonary disease, and chronic anemia [3].

Kidney disease is among the most common and serious complications in this group. Sickle cell nephropathy, driven by recurrent medullary ischemia, papillary necrosis, and progressive glomerulosclerosis, is a major cause of chronic kidney disease and eventual end-stage renal disease (ESRD) [4]. The coexistence of ESRD with SCD significantly complicates management, as hemodialysis introduces additional challenges in fluid balance, nutrition, and medication dosing [5]. While kidney problems are expected in many aging SCD patients, less common blood disorders can also appear and further complicate the picture.

Autoimmune hemolytic anemia (AIHA) is an uncommon but potentially life-threatening hematologic disorder characterized by the immune-mediated destruction of red blood cells. In SCD, AIHA is especially rare and challenging to diagnose, as the baseline chronic hemolysis characteristic of the disease can obscure its recognition [6]. Mixed AIHA, marked by the presence of both warm (IgG) and cold (IgM) autoantibodies, is even more uncommon and often associated with a more severe and refractory clinical course compared to warm or cold AIHA alone [7].

The diagnosis and management of mixed AIHA in a patient with SCD is further complicated by transfusion-related alloimmunization, a common problem in patients with chronic transfusion exposure. Alloantibody formation can severely limit access to compatible blood, delay transfusion, and increase the risk of transfusion reactions [8]. In elderly patients, comorbidities such as ESRD and prior malignancy further narrow therapeutic options and increase the risk of treatment-related complications.

This report highlights the diagnostic complexity and therapeutic challenges of mixed AIHA in an elderly man with homozygous SCD and ESRD, emphasizing the importance of early recognition and individualized care in this high-risk population.

## Case presentation

A male patient in his late 60s with a history of homozygous SCD and ESRD on a thrice-weekly hemodialysis schedule presented with fatigue and a significant drop in hemoglobin (HgB), identified during routine dialysis. He denied overt bleeding, melena, or symptoms of vaso-occlusive crisis. Despite adherence to hydroxyurea and folic acid, the patient had a chronic history of progressive anemia from SCD, with his baseline HgB averaging around 7.5 g/dL over the past year. His medical history also included paroxysmal atrial fibrillation and prostate cancer, treated with radiation therapy. There was no history of autoimmune disease or previous immune therapy.

Initial laboratory evaluation revealed a HgB of 4.5 g/dL, hematocrit (Hct) of 13.4%, lactate dehydrogenase (LDH) of 231 U/L, and a reticulocyte count of 8.31%, suggesting active hemolysis (Table 1). Given the severity and unclear etiology of anemia, an autoantibody panel was obtained and revealed the presence of both warm (IgG-mediated) and cold (IgM-mediated) autoantibodies, consistent with a diagnosis of mixed AIHA, a rare finding in patients with SCD.

**Table 1:** Laboratory values on admission HgB, hemoglobin; Hct, hematocrit; LDH, lactate dehydrogenase

	Reference range	Admission	Interpretation
HgB (g/dL)	13.5 - 17.5	4.5	Severely reduced
Hct (%)	41.0 - 53.0	13.4	Severely reduced
LDH (U/L)	100 - 190	231	Mildly elevated
Reticulocyte count (%)	0.60 - 2.20	8.31	Markedly elevated

Transfusion efforts were complicated by crossmatch incompatibility due to underlying alloimmunization. The blood bank identified phenotypically matched, leukoreduced, HgB S-negative red blood cells that were least incompatible. Corticosteroid therapy, with prednisone at 1 mg/kg daily, was initiated to suppress the autoimmune process.

Nephrology managed volume status carefully during dialysis, given peripheral edema and limited oral intake. The patient also had secondary hyperphosphatemia, for which phosphate binders were restarted. Nutritional assessment revealed protein-calorie malnutrition, prompting a high-protein renal diet and supplemental support.

Following initiation of steroid therapy and continued hemodialysis, the patient’s HgB improved to 7.2 g/dL. He was discharged in stable condition, with plans for close outpatient hematology and nephrology follow-up.

## Discussion

Mixed AIHA is an especially rare form of autoimmune hemolysis, comprising less than 10% of all AIHA cases [9]. It is defined by the simultaneous presence of warm autoantibodies (usually IgG, active at 37°C) and cold autoantibodies (typically IgM, active below 30°C), which act through distinct immune mechanisms to promote red blood cell destruction [10]. Mixed AIHA is most commonly associated with underlying lymphoproliferative disorders (such as chronic lymphocytic leukemia and non-Hodgkin lymphoma), systemic autoimmune diseases (including systemic lupus erythematosus and rheumatoid arthritis), and infections (notably viral infections like HIV and SARS-CoV-2); however, a proportion of cases are idiopathic, with no identifiable underlying cause [11]. Reports of AIHA in patients with SCD are exceedingly uncommon. In the case series and literature review by Motta et al., AIHA was identified as a rare complication of congenital anemias, though only one case of warm AIHA was documented in a patient with isolated SCD [6]. More recently, a refractory case of warm AIHA in a sickle cell patient was successfully managed with bortezomib [12]. To our knowledge, however, there are no previously documented cases of mixed AIHA in patients with SCD, making this presentation particularly rare and noteworthy.

The coexistence of SCD and AIHA presents several diagnostic challenges. Patients with SCD typically have chronic hemolysis at baseline, with elevated LDH and reticulocyte counts, making it difficult to recognize superimposed autoimmune hemolysis without specific antibody testing [6]. Furthermore, many SCD patients have a history of chronic transfusion, predisposing them to alloimmunization and the development of antibodies against foreign red cell antigens [13]. These alloantibodies, alongside autoantibodies, create a highly complex immunohematologic profile that complicates crossmatching, delays transfusion, and increases the risk of hemolytic transfusion reactions.

In this case, transfusion support was hindered by multiple incompatible serologic reactions, despite the use of phenotypically matched, leukoreduced, HgB S-negative units. The delay in obtaining compatible blood placed the patient at risk for cardiovascular decompensation, highlighting the critical need for advance planning and coordination with transfusion medicine in patients with complex antibody profiles.

Therapeutically, corticosteroids remain the cornerstone of initial treatment for AIHA, including mixed AIHA. However, response to steroids is often incomplete in mixed cases, and additional immunosuppressive therapy, such as rituximab or cytotoxic agents, is frequently required [14]. In elderly patients with comorbidities like ESRD, the use of such agents carries significant risks, including immunosuppression, infection, and poor wound healing [15]. The American Society of Hematology currently suggests immunosuppression in all patients with a high risk of hemolytic transfusion reaction and acute need for transfusion [16]. However, further studies are required to better characterize the patients for whom immunosuppression is most appropriate. The need for dialysis further complicates fluid management, nutritional support, and medication dosing.

The age of this patient is also a key point of clinical interest. Most individuals with homozygous SCD do not live into their late 60s, and the presence of this autoimmune complication at such an advanced age is exceedingly rare. As survival improves in the SCD population, clinicians should be aware of the potential for atypical hematologic manifestations in older adults, including autoimmune cytopenias like AIHA.

## Conclusions

Mixed AIHA in an elderly patient with homozygous SCD represents an exceptionally rare and diagnostically complex condition. The coexistence of warm and cold autoantibodies created major transfusion challenges in the setting of alloimmunization, while advanced age, ESRD, and multiple comorbidities further narrowed therapeutic options and increased the risk of complications. This case demonstrates that even in patients with chronic hemolysis from SCD, clinicians should consider superimposed autoimmune processes when faced with an acute and profound decline in HgB.

Early antibody screening, advanced coordination with transfusion services, and individualized immunosuppressive strategies are critical for managing these high-risk patients. As survival improves in the SCD population, similar presentations may become more frequent, highlighting the importance of vigilant recognition and multidisciplinary care to achieve favorable outcomes.
